# Procalcitonin and C-reactive protein as diagnostic biomarkers in COVID-19 and Non-COVID-19 sepsis patients: a comparative study

**DOI:** 10.1186/s12879-023-08962-x

**Published:** 2024-01-04

**Authors:** Jing Shi, Ying Zhuo, Ting-Qiang Wang, Chun-E Lv, Ling-Hui Yao, Shi-Yan Zhang

**Affiliations:** 1 Department of Anesthesiology, Fuding Hospital, Fujian University of Traditional Chinese Medicine, Fuding, Fujian 355200 China; 2 Department of Clinical Laboratory, Fuding Hospital, Fujian University of Traditional Chinese Medicine, Fuding, Fujian 355200 China

**Keywords:** COVID-19, Sepsis, Procalcitonin, C-reactive protein, Biomarkers, Gram-positive bacteria, Gram-negative bacteria

## Abstract

**Background:**

This study aimed to assess and compare procalcitonin (PCT) and C-reactive protein (CRP) levels between COVID-19 and non-COVID-19 sepsis patients. Additionally, we evaluated the diagnostic efficiency of PCT and CRP in distinguishing between Gram-positive (GP) and Gram-negative (GN) bacterial infections. Moreover, we explored the associations of PCT with specific pathogens in this context.

**Methods:**

The study included 121 consecutive sepsis patients who underwent blood culture testing during the COVID-19 epidemic. PCT and CRP were measured, and reverse transcriptase-polymerase chain reaction (RT-PCR) was employed for the detection of COVID-19 nucleic acid. The Mann-Whitney U-test was used to compare PCT and CRP between the COVID-19 and non-COVID-19 groups. Receiver operating characteristic (ROC) curves were generated to compare PCT and CRP levels in the GN group versus the GP group for assessing the diagnostic efficiency. The kruskal-Wallis H test was applied to assess the impact of specific pathogen groups on PCT concentrations.

**Results:**

A total of 121 sepsis patients were categorized into a COVID-19 group (n = 25) and a non-COVID-19 group (n = 96). No significant differences in age and gender were observed between the COVID-19 and non-COVID-19 groups. The comparison of biomarkers between these groups showed no statistically significant differences. The optimal cut-off values for PCT and CRP in differentiating between GP and GN infections were 1.03 ng/mL and 34.02 mg/L, respectively. The area under the ROC curve was 0.689 (95% confidence interval (CI) 0.591–0.786) for PCT and 0.611 (95% CI 0.505–0.717) for CRP. The diagnostic accuracy was 69.42% for PCT and 58.69% for CRP. The study found a significant difference in PCT levels among specific groups of pathogens (*P* < 0.001), with the highest levels observed in *Escherichia coli* infections. The frequency of *Staphylococcus spp.* positive results was significantly higher (36.0%) in COVID-19 compared to non-COVID-19 sepsis patients (*P* = 0.047).

**Conclusion:**

Sepsis patients with COVID-19 revealed a significantly higher culture positivity for *staphylococcus spp.* than the non-COVID-19 group. Both PCT and CRP showed moderate diagnostic efficiency in differentiating between GP and GN bacterial infections. PCT showed potential utility in identifying *E. coli* infections compared to other pathogens.

## Introduction

Sepsis is a life-threatening organ dysfunction caused by a dysregulated host response to infection, resulting in high morbidity and mortality rates worldwide [1]. Early diagnosis and appropriate treatment of sepsis are crucial for improving patient outcomes [2]. The coronavirus disease 2019 (COVID-19) pandemic has posed additional challenges in the management of sepsis due to the similarities in clinical presentation and the potential for bacterial coinfections [3]. Biomarkers such as procalcitonin (PCT) and C-reactive protein (CRP) have been widely used to aid in the diagnosis and management of sepsis, as they are known to increase in response to bacterial infections [4, 5].

Procalcitonin is a peptide precursor of calcitonin, which is produced by the C-cells of the thyroid gland. Its serum levels rise rapidly in response to bacterial infections and can help differentiate between bacterial and viral infections [6, 7]. Although elevated PCT serum concentrations are not exclusive to infections, PCT is still considered to be one of the best biomarkers available to diagnose sepsis [8]. CRP is an acute-phase protein synthesized by the liver in response to inflammation and tissue damage. It is a nonspecific marker of inflammation, but its levels can be significantly elevated in bacterial infections compared to viral infections [9, 10].

However, our knowledge about the specific PCT and CRP levels associated with distinct pathogens in COVID-19 sepsis patients remains limited. Notably, there has been no prior investigation into the comparative frequency and microbiological characteristics of pathogen distribution in COVID-19 sepsis concerning PCT and CRP levels, particularly in the context of China. This study aimed to compare PCT and CRP levels between sepsis patients with COVID-19 and those without, to evaluate the diagnostic efficiency of PCT and CRP in distinguishing between Gram-positive (GP) and Gram-negative (GN) bacterial infections, and to analyze whether particular pathogens have a relevant impact on serum concentrations of PCT.

## Materials and methods

### Study design and participants

This retrospective study included 25 patients with COVID-19 sepsis and 96 patients with non-COVID-19 sepsis admitted to Fuding Hospital, Fujian University of Traditional Chinese Medicine, between January and December 2022. Diagnosis of COVID-19 sepsis was based on a positive reverse transcriptase-polymerase chain reaction (RT-PCR) test for SARS-CoV-2 (severe acute respiratory syndrome coronavirus 2) [11]. The presence of sepsis was determined according to the Third International Consensus Definitions for Sepsis and Septic Shock (Sepsis-3) [12]. Non-COVID-19 control subjects were patients diagnosed with sepsis without COVID-19. The study protocol received approval from Medical Ethics Committee of Fuding Hospital, Fujian University of Traditional Chinese Medicine (ethical approval number: Fuding Hospital 2,023,001). Written informed consent was waived by Medical Ethics Committee of Fuding Hospital, Fujian University of Traditional Chinese Medicine, due to the retrospective nature of the study.

### Data collection

Peripheral venous puncture was employed to collect the patient’s blood, which was then transferred into a Bactec vial prior to antibiotic treatment. Subsequently, the samples were underwent incubation in a Bactec incubator (BD Diagnostics, Franklin Lakes, NJ, USA) until the results were obtained. Only isolates meeting predefined criteria for pathogenicity were included in the analysis. Pathogens were categorized into two major groups: Gram-positive and Gram-negative bacteria. Within each category, pathogens were further divided into five distinct phylogenetic groups, namely *Staphylococcus spp., Streptococcus spp., Enterococcus spp., E coli*, and *Klebsiella pneumonia.* A novel sixth group was created to categorize rarely detected pathogens that did not align with the existing five groups, based on specific criteria. Additionally, *Staphylococcus spp*. included *Staphylococcus aureus, Staphylococcus epidermidis, Staphylococcus saprophyticus*, and *Staphylococcus haemolyticus. Enterococcus spp* comprised *Enterococcus faecalis, Enterococcus faecium, Enterococcus gallinarum*, and *Enterococcus avium*.

Before initiating anti-infective therapy upon admission, a nasopharyngeal swab underwent testing for coronavirus nucleic acid by RT-PCR (real-time polymerase chain reaction) technology. Xi’an Tianlong Gene Co., Ltd. supplied the reagents, positive and negative controls, an automated nucleic acid extractor, and a fluorescent PCR instrument. Results were interpreted as positive when the Cycle threshold (Ct) values of N gene (virus nucleocapsid) and ORF1ab gene (open reading frame 1a and 1b) below 40, in accordance with the latest guideline in China (Trial 9th version) [13].

Clinical parameters, such as age, gender, and laboratory data, were retrieved from electronic medical records. Biomarkers, including PCT, CRP, white blood cell count (WBC), total protein (TP), albumin protein (ALB), platelet count (PLT), red blood cell distribution width (RDW), and neutrophil-lymphocyte count ratio (NLR), were measured within 24 h of admission.

Serum PCT levels were measured using the Cobas e411/E601 (Roche Diagnostics, Mannheim, Germany). The Dimension Vista 1500 Intelligent Lab system (Siemens Healthcare GmbH, Erlangen, Germany) was utilized to analyze CRP following the manufacturer’s instructions. A fully automated clinical chemistry analyzer (Beckman Coulter AU 5800, USA) was utilized to measure TP and ALB. Additionally, the Sysmex XN-9000 hematology analyzer (Sysmex Corporation, Kobe, Japan) was employed to determine the complete blood cell count, WBC, PLT, RDW, and calculated NLR.

### Statistical analysis

Statistical analysis was carried out using the Statistical Package for Social Sciences (SPSS) Version 22.0 (Chicago IL, USA) and GraphPad Prism 8.0 (GraphPad software, San Diego California USA, www.GraphPad. com). The Kolmogorov-Smirnov test was used to assess the distribution of PCT and CRP concentrations in both groups, providing median values and interquartile ranges (IQR). The Chi-square test or Fisher’s exact test, as appropriate, was employed to compare categorical data or proportions between the COVID-19 and non-COVID-19 groups. The Mann-Whitney U-test, a nonparametric statistical test, was applied to compare two independent samples when the data could not be assumed to be normally distributed, determining if there was a significant difference between the mean ranks of the two groups. The Kruskal-Wallis H test, suitable for comparing more than two subgroups when the data cannot be assumed to be normally distributed, was employed to assess differences among multiple subgroups.

Receiver operating characteristic (ROC) curves, which correlated true-positive (sensitivity) and false-positive (1-specificity) rates, were constructed to analyze diagnosis value of CRP and PCT for the predication of GN infections. These ROC curves were constructed, and the optimal cutoff values were determined using Youden’s index. Various metrics, including sensitivity, specificity, positive predictive value, negative predictive value, positive likelihood ratio, negative likelihood ratio, and accuracy, were calculated to evaluate diagnostic performance. Additionally, the Diagnostic Odds Ratio (DOR) was used as a measure of effectiveness. DOR represents the ratio of the odds of a positive test in patients with the disease to the odds of a positive test in patients without the disease [14]. In all tests, statistical significance was considered at a *P* value less than or equal to 0.05 (*P* ≤ 0.05).

## Results

### Clinical parameters of study population

The current study, comprising 121 patients who underwent blood culture tests at Fuding Hospital, including 25 patients in the COVID-19 group and 96 in the non-COVID-19 group. The characteristics of the patients included in the study are listed in Table 1. The median age of COVID-19 patients was 71.0 years (Interquartile Range (IQR): 51.0–79.5), while that of CNT was 66.0 years (IQR: 49.5–73.8). However, the difference was not statistically significant (*P* = 0.158). The gender distribution was similar between the two groups (*P* = 0.601).

**Table 1:** Comparison of age and gender distribution between COVID-19 group and non-COVID-19 group [number (%)]

Item	COVID-19 (n = 25)	non-COVID-19 (n = 96)	Mann-Whitney *U*-test /*χ*^*2*^	*P* value
Age Median (IQR) (years)	71.0 (51.0–79.5)	66.0 (49.5–73.8)	-1.412	0.158
Gender, n (%)			0.273	0.601
Male	15 (60.0)	52 (54.2)		
Female	10 (40.0)	44 (45.8)		

IQR, interquartile range

### Kolmogorov-sminov test

To analyze the statistical distribution of each marker tested in each group, a Kolmogorov-Smirnov test was conducted. The results showed that the ORF and N markers had a normal distribution, with a mean cycle threshold value (CT) of ORF gene (26.94 ± 5.49) and N gene (24.29 ± 4.95), respectively, and a Z score range of 0.138–0.140 and a *P* -value of 0.200. However, the results of the other groups indicated a non-normal distribution, with a Z score range of 0.081 to 0.368 and a *P* - value of less than 0.01. Therefore, non-parametric tests were used to conduct subsequent statistical analyses.

### Laboratory parameters

The results of the laboratory parameters are presented in Table 2. The biomarker levels of the COVID-19 group were compared with those of the non-COVID-19 group, revealing no significant differences in PCT and CRP as well as TP, ALB, WBC, PLT, RDW, or NLR between the two groups.

**Table 2:** Comparison of biomarkers between COVID-19 group and non-COVID-19 group (IQR).

Item	COVID-19 (n = 25)	non-COVID-19 (n = 96)	*Z*	*P* value
CRP (mg/L)	43.88 (7.76–148.80)	87.69 (43.21–140.05)	-1.947	0.051
PCT (ng/mL)	0.89 (0.10–74.21)	3.12 (0.27–17.45)	-0. 490	0.624
TP (g/L)	60.00 (53.05–64.20)	61.00 (55.43–67.30)	-1.402	0.161
ALB (g/L)	30.80 (28.50–33.95)	32.05 (28.88–36.95)	-0.615	0.539
WBC (10^9^/L)	9.23 (7.24–11.75)	8.51 (6.11–12.16)	-0.333	0.739
Neutrophiles (10^9^/L)	8.11 (4.17–11.11)	6.48 (3.67–10.88)	-0.653	0.514
Lymphocytes (10^9^/L)	0.62 (0.34–1.33)	0.89 (0.56–1.47)	-1.748	0.080
PLT (10^9^/L)	174.00 (103.50–273.00)	170.50 (98.50–249.00)	-0.285	0.776
RDW (%)	13.20 (12.70–15.00)	14.00 (13.00–15.00)	-0.663	0.507
NLR	12.18 (4.98–35.17)	7.67 (3.48–14.38)	-1.620	0.105

Mann-Whitney U-test was carried out to compare results between the two groups. IQR, interquartile range; PCT, procalcitonin; CRP, C-reactive protein; WBC, white blood cell count; TP, total protein; ALB, albumin protein; PLT, platelet count; RDW, red blood cell distribution width; NLR, neutrophil-lymphocyte count ratio

### Biomarkers in GP and GN bacterial infections

Table 3 compares biomarkers in GP and GN bacterial infections. CRP and PCT levels were higher in GN infections (*P* = 0.038 and *P* < 0.001, respectively), with no significant differences in TP, ALB, WBC, PLT, or NLR.

**Table 3:** Comparison of biomarkers between GP group and GN one (IQR)

Item	GP (n = 49)	GN (n = 72)	Z	*P* value
CRP (mg/L)	59.50 (10.41–125.26)	90.82 (48.25–149.72)	-2.070	**0.038**
PCT (ng/mL)	0.46 (0.15–6.80)	8.08 (0.95–24.80)	-3.514	**< 0.001**
TP (g/L)	60.60 (55.55–65.60)	60.65 (54.70–62.28)	-0.119	0.905
ALB (g/L)	30.70 (28.60–34.30)	32.25 (29.83–37.23)	-1.362	0.173
WBC (10^9^/L)	8.44 (5.46–11.92)	9.26 (6.54–12.39)	-0.908	0.364
Neutrophiles (10^9^/L)	6.12 (3.32–10.20)	7.89 (4.46–11.27)	-1.064	0.287
Lymphocytes (10^9^/L)	0.86 (0.51–1.34)	0.86 (0.52–1.44)	-0.011	0.992
PLT (10^9^/L)	181.00 (110.00–293.50)	167.00 (88.00–242.00)	-1.489	0.136
RDW (%)	14.00 (13.00–16.00)	14.00 (13.00–15.00)	-0.779	0.436
NLR	7.52 (3.33–14.53)	8.33 (3.69–16.85)	-0.808	0.419

Mann-Whitney U-test was carried out to compare results between GP and GN group. IQR, interquartile range; GP, Gram-Positive; GN, Gram-Negative; PCT, procalcitonin; CRP, C-reactive protein; WBC, white blood cell count; TP, total protein; ALB, albumin protein; PLT, platelet count; RDW, red blood cell distribution width; NLR, neutrophil-lymphocyte count ratio

### Prediction of Gram-negative bacterial Infections

Blood cultures were positive in 121 sepsis patients, including 49 patients with GP and 72 with GN bacterial infections.

**Fig. 1:**
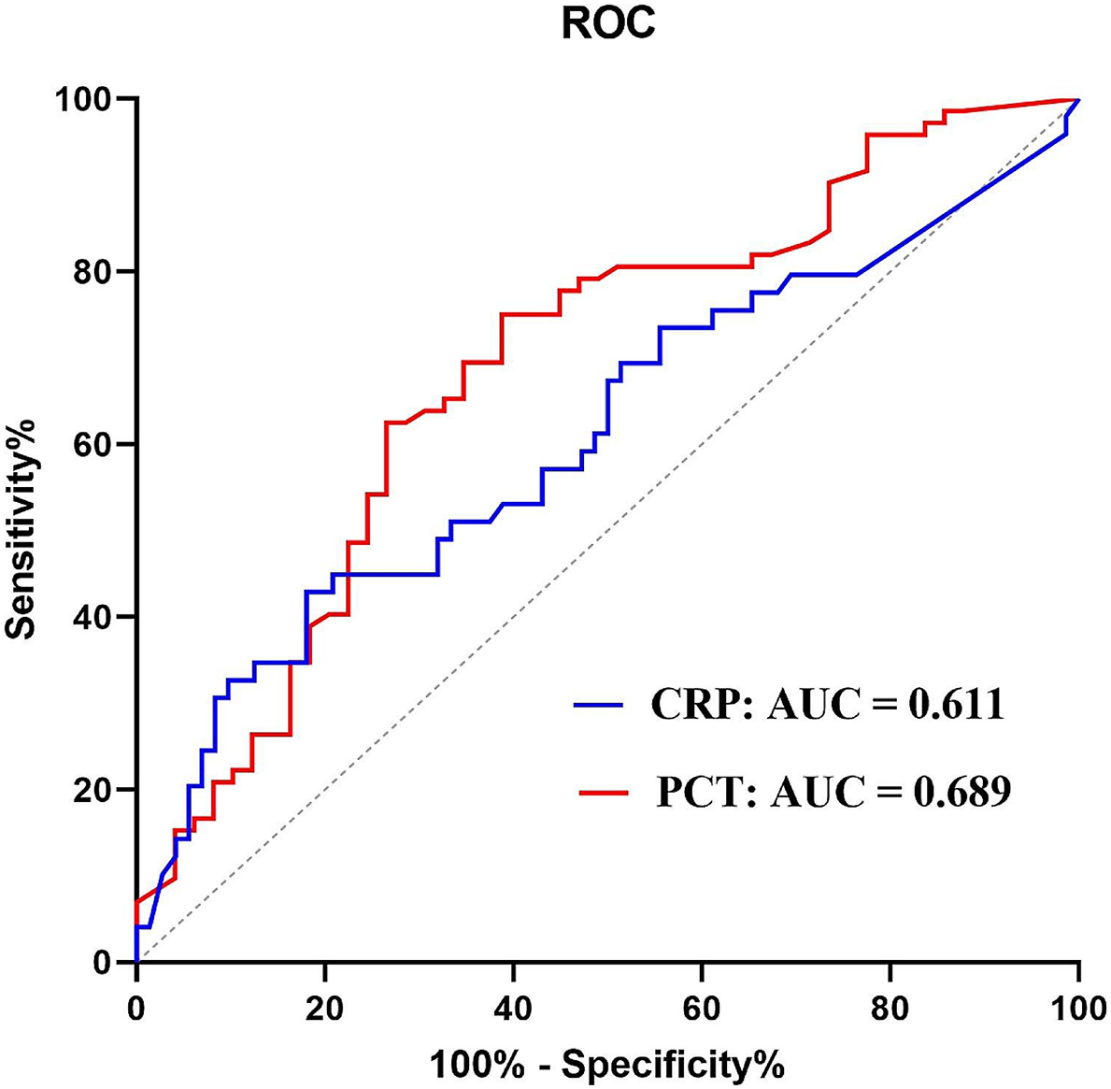
ROC curve comparing PCT and CRP levels in GN group versus GP group. The curves show optimal cut-off value for PCT of 1.03 ng/mL and for CRP of 24.02 mg/L. PCT, procalcitonin; CRP, C-reactive protein

The study constructed ROC curves to analyze PCT and CRP in the GN group compared to the GP group. The AUC of PCT and CRP were 0.689 (95% confidence interval (CI) 0.591–0.786) and 0.611 (95% CI 0.505–0.717), respectively. Sensitivity, specificity, Youden’s index (sensitivity + specificity − 1), positive predictive value, negative predictive value, positive likelihood ratio, negative likelihood ratio, diagnostic odds ratio (DOR) and diagnostic accuracy were calculated for PCT and CRP in accordance with the ROC curves, as shown in Table 4; Fig. 1.

**Table 4:** The diagnosis efficiency of PCT and CRP (GN group versus GP group)

Item	PCT	CRP
Optimal cutoff	1.03 ng/mL	34.02 mg/L
AUC (95% CI)	0.689 (0.591–0.786)	0.611 (0.505–0.717)
Standard error	0.050	0.054
Sensitivity, % (95% CI)	75.00 (63.91–83.56)	42.86 (30.02–56.73)
Specificity, % (95% CI)	61.22 (47.25–73.57)	81.94 (71.52–89.13)
Diagnostic accuracy, % (95% CI)	69.42(57.16–79.51)	58.69 (46.83–69.85)
Positive predictive value, % (95% CI)	73.97 (64.03–82.29)	77.71 (60.77–88.46)
Negative predictive value, % (95% CI)	62.50 (47.12–75.28)	49.39 (41.02–58.37)
Positive likelihood ratio, (95% CI)	1.93 (1.21–3.16)	2.37 (1.05–5.22)
Negative likelihood ratio, (95% CI)	0.41 (0.22–0.76)	0.70(0.49–0.98)
Diagnostic odds ratio, (95% CI)	4.73 (1.59–14.14)	3.40 (1.08–10.75)
Youden’s index (%)	36.22	24.80
*P* value	< 0.001	0.039

AUC, area under the receiver operating characteristic. CI, confidence interval. PCT, procalcitonin. CRP, C-reactive protein

### Distribution of microorganisms

In COVID-19 sepsis patients, *E. coli* (40.0%) and *Staphylococcus spp.* (36.0%) were the primary bacterial findings, while the control group exhibited *E. coli* (37.5%) and *K. pneumoniae* (18.8%). *Staphylococcus spp*. was significantly more common in COVID-19 (36.0%) than in non-COVID-19 sepsis patients (*P* = 0.047) (Table 5).

**Table 5:** Frequency of bacterial pathogens isolated from blood cultures in COVID-19 and non-COVID-19 sepsis patients (%)

Microorganisms		COVID-19 (n = 25)	non-COVID-19 (n = 96)	Chi-square	*P* value	
Gram-positive bacteria (%)		13 (52.0)	36 (37.5)	1.731	0.188	
1, *Staphylococcus spp.*		9 (36.0)	17 (17.7)	3.934	0.047	
	*Staphylococcus aureus*	1	6			
	*Staphylococcus hominis*	1	3			
	*Streptococcus mitis*	1	4			
	*Staphylococcus epidermidis*	6	4			
2, *Streptococcus spp.*		2 (8.0)	15 (15.6)	0.428	0.513	
	*Streptococcus pneumoniae*		6			
	*Streptococcus sanguinis*		1			
	*Streptococcus agalactiae*		1			
	*Neisseria gonorrhoeae*		1			
	*Streptococcus milleri*		1			
	*Streptococcus pyogenes*	1	2			
	*Streptococcus salivarius*		1			
	*Streptococcus intermedius*		2			
	*Streptococcus adjacens*	1	0			
3, *Enterococcus spp.*		2 (8.0)	4 (4.2)	-	0.602*	
	*enterococcus faecium*	1	0			
	*Enterococcus faecalis*	1	4			
Gram-negative bacteria		12 (48.0)	60 (62.5)			
4,	*Escherichia coli*	10 (40.0)	36 (37.5)	0.053	0.819	
5,	*Klebsiella pneumoniae*	2 (8.0)	18 (18.8)	0.974	0.324	
6,	Rare pathogens		6			

*: Fisher’s exact test. Rare pathogens (each with only one reported strain): *Neisseri elongata, Proteus mirabilis, Aeromonas hydrophila, Salmonella enterica, Pseudomonas aeruginosa, Moraxella osloensis*

**Fig. 2:**
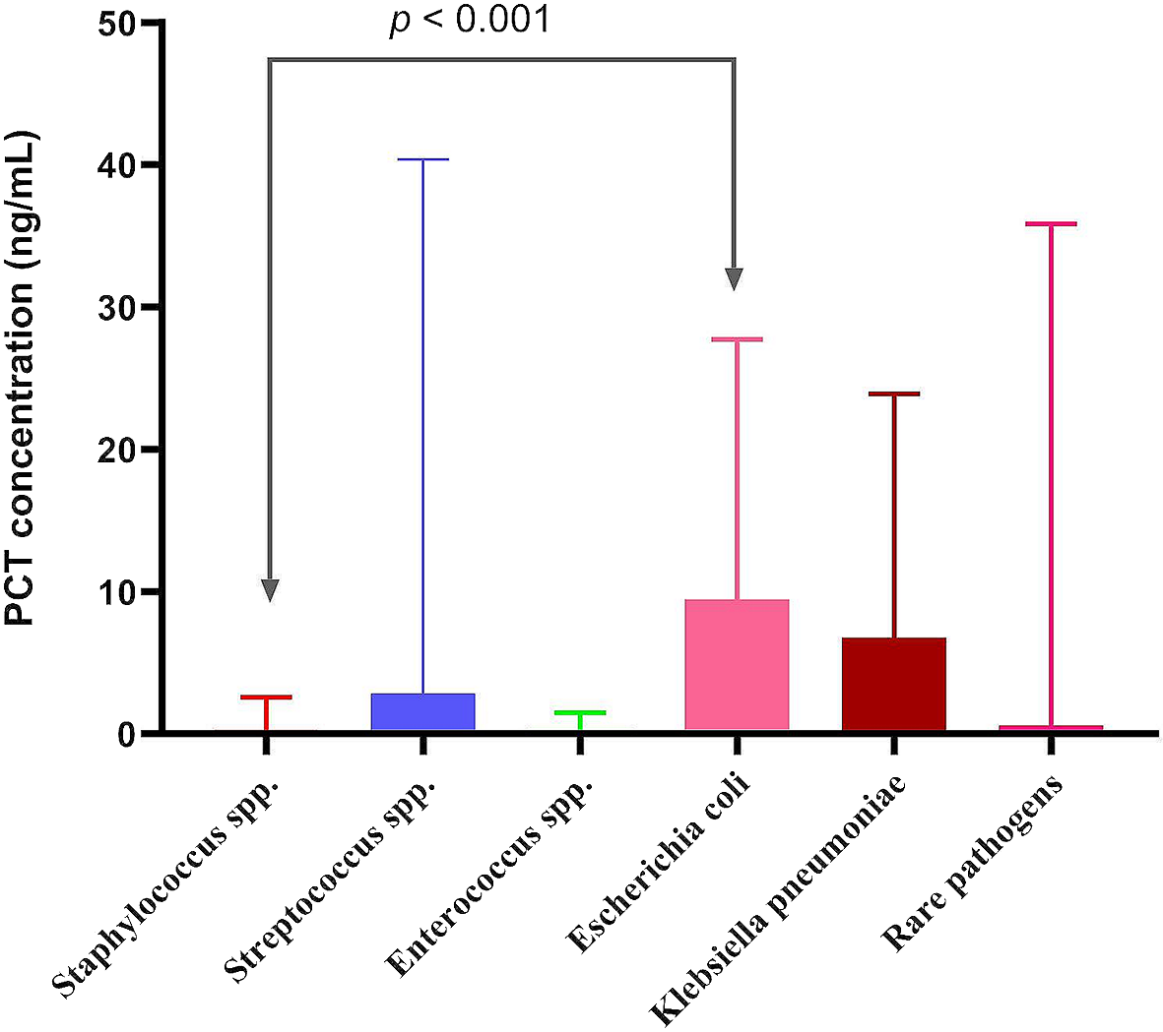
PCT concentrations (median, ng/mL) associated with type of pathogen. *Escherichia coli* had the highest PCT (9.43 ng/mL), *Klebsiella pneumoniae* followed (6.76 ng/mL), and *Staphylococcus spp*. had the lowest (0.28 ng/mL), showing significant differences (*P* < 0.001). PCT, procalcitonin; CRP, C-reactive protein; Rare pathogens (*Neisseri elongata, Proteus mirabilis, Aeromonas hydrophila, Salmonella enterica, Pseudomonas aeruginosa, Moraxella osloensis.* each with only one reported strain)

PCT levels varied significantly among sepsis patients with different bacterial infections. *Staphylococcus spp*. had the lowest median PCT levels (0.28 ng/mL), while *Enterococcus spp.* had a median of 0.29 ng/mL. In contrast, *E. coli* had the highest median PCT levels (9.43 ng/mL), followed by *K. pneumoniae* (6.76 ng/mL). Patients with GN infections from *E. coli* had significantly higher PCT values than those from *Staphylococcus spp.* (9.43 vs. 0.28 ng/mL, *P* < 0.001). No significant PCT differences were observed among bacteremia caused by *Staphylococcus spp*., *Streptococcus spp*., *Enterococcus spp*., *K. pneumoniae*, and rare pathogens (*Neisseri elongata, Proteus mirabilis, Aeromonas hydrophila, Salmonella enterica, Pseudomonas aeruginosa, Moraxella osloensis.* Each with only one strain) (Tables 5 and 6) (Fig. 2).

**Table 6:** Procalcitonin values associated with different pathogens

Bacteria	Number	PCT (IQR) (ng/mL)	Kruskal-Wallis H	*P* value
*Staphylococcus spp.*	26	0.28 (0.14–2.61)	21.959	< 0.001
*Streptococcus spp.*	17	2.85 (0.22–40.39)		
*Enterococcus spp.*	6	0.29 (0.05–1.49)		
*Escherichia coli*	46	9.43 (1.50–27.72)		
*Klebsiella pneumoniae*	20	6.76 (1.06–23.91)		
Rare pathogens	6	0.58 (0.13–35.83)		

Kruskal-Wallis H test was performing to assess differences among the six subgroups. Statistically significant differences (*P* < 0.001) were found in the overall distribution of the data. Then, the post-hoc pairwise comparisons were conducted using Dunn’s test, which exhibited statistically significant differences between *Staphylococcus spp.* and *Escherichia coli* (*H* = − 3.874, *P* < 0.001). IQR, interquartile range; PCT, procalcitonin

## Discussion

Our study explored the impact of COVID-19 and other pathogens affected PCT and CRP in sepsis patients. We observed no statistically significant differences in PCT and CRP levels between COVID-19 and non-COVID-19 groups. *Staphylococcus spp*. was more prevalent (36.0%) in COVID-19 patients, and PCT seemed to be more effective in identifying *E. coli* infections.

In the comparison of PCT and CRP between the GP and GN groups, we observed significant differences in PCT and CRP levels. The optimal cut-off values for PCT and CRP in distinguishing between these infections were determined to be 1.03 ng/mL and 34.02 mg/L, respectively. Notably, the diagnostic efficiency of PCT was higher than that of CRP. The results presented in Table 2 revealed that PCT and CRP concentrations were markedly higher in the GN group than the GP group. The ROC curve illustrated in Fig. 1 demonstrated that PCT possessed a sensitivity of 75.00%, a specificity of 66.22%, and diagnostic accuracy of 69.42% when distinguishing between GN and GP. This was consistent with prior findings by Daniel O. Thomas-Rüddel et al. [15], who reported a PCT sensitivity of 69.0% and specificity of 65.0%. Several researchers have also suggested that extremely elevated PCT concentrations are linked to GN bacteremia, supporting the idea of tailoring antimicrobial therapy based on PCT levels [16, 17]. Variations in PCT concentrations may be linked to pathogen-specific signaling, as inflammatory cytokines partially induce PCT expression [18]. Lipopolysaccharides (LPS), which are cell wall components of GN bacteria, represent the prototypical class of pathogen-associated molecular patterns (PAMPs) [19]. They are recognized by cells of the innate immune system through toll-like receptor 4 (TLR4) [19]. However, the TLR4-dominant activation by GN bacteria results in a distinctly different induction of several inflammatory cytokines, potentially contributing to the observed differences in PCT response seen with GN.

The study highlighted the importance of concurrently measuring PCT and CRP for the precise detection of GN bacteremia. Therefore, utilizing PCT alongside other diagnostic tools could enhance the accuracy of diagnosing and treating bacterial infections [20].

The distribution of microorganisms in COVID-19 sepsis patients and non-COVID-19 control subjects was generally similar, except for *Staphylococcus spp*. This finding implied that the etiology of sepsis in COVID-19 patients might be similar to that in non-COVID-19 control subjects. However, the significantly elevated PCT values observed in *E. coli* infections compared to *Staphylococcus spp*. infections suggested that PCT levels might be useful in identifying the causative pathogen in sepsis patients, particularly in distinguishing between these two common bacterial infections. These findings were consistent with the results reported by Thomas-Rüddel DO, et al., who observed that PCT concentrations differed significantly between specific pathogens groups (*P* < 0.001), with the highest concentrations in *E. coli, Streptococcus* species, and other *Enterobacteriaceae* [15].

Our study has some limitations. Firstly, the sample size was relatively small, potentially limited the statistical power of our findings. Secondly, the retrospective nature of the study might have introduced some bias in data collection and analysis. Finally, our study population comprised patients from a single center, possibly limiting the generalizability of our findings to other settings. To resolve these limitations, further studies with larger sample sizes and multi-center designs are needed to confirm our findings and explore the potential clinical applications of these biomarkers in the management of sepsis patients.

## Conclusion

Our study highlighted potential utility of PCT and CRP levels for differentiation between COVID-19 and non-COVID-19 sepsis patients, as well as for distinguishing between GP and GN bacterial infections in sepsis patients. Notably, PCT exhibited higher diagnostic efficiency compared to CRP, suggesting its potential as a more reliable biomarker for the differentiation of these infections. Moreover, PCT showed particular promise in identifying *E. coli* infections compared to other pathogens.

## Data Availability

The datasets used and/or analyzed during the current study available from the corresponding author upon reasonable request.

## Abbreviations

PCT: Procalcitonin
CRP: C-reactive Protein
COVID-19: Coronavirus disease 2019
GN: Gram-Negative
GP: Gram-Positive
RT-PCR: Reverse transcriptase-polymerase chain reaction
SARS-CoV-2: Severe acute respiratory syndrome coronavirus 2
WBC: White blood cell count
TP: Total protein
ALB: Albumin protein
RDW: Red blood cell distribution width
NLR: Neutrophil-lymphocyte count ratio
IQR: Interquartile ranges
ROC: Receiver operating characteristic
CI: Confidence interval
AUC: Area under receiver operating characteristic curve
DOR: Diagnostic odds ratio
LPS: Lipopolysaccharides
PAMPs: Pathogen-associated molecular patterns
TLR4: Toll-like receptor 4
